# Dulaglutide improves glucocorticoid-induced hyperglycemia in inpatient care and reduces dose and injection frequency of insulin

**DOI:** 10.1186/s12902-020-0542-5

**Published:** 2020-05-07

**Authors:** Hiroyuki Uchinuma, Masashi Ichijo, Noriyuki Harima, Kyoichiro Tsuchiya

**Affiliations:** Department of Diabetes and Endocrinology, University of Yamanashi Hospital, 1110 Shimokato, Chuo, Yamanashi, 4093898 Japan

**Keywords:** Dulaglutide, Glucocorticoid-induced hyperglycemia, Diabetes mellitus, Insulin

## Abstract

**Background:**

Glucocorticoid (GC)-induced hyperglycemia is characterized by elevated postprandial blood glucose, which commonly requires multiple insulin injections. We investigated whether a long-acting glucagon-like peptide-1 receptor agonist, dulaglutide (Dula), safely improved GC-induced hyperglycemia in inpatients, to reduce insulin injection frequency.

**Methods:**

The data of hospitalized patients with GC-induced hyperglycemia treated with Dula (Dula group, *n* = 38) or without (non-Dula group, n = 38) were retrospectively evaluated. Baseline data were collected at the beginning of GC treatment. The primary outcome in this study was glycemic control, which was compared between the groups using the six-point blood glucose (before and 2 h after each meal) profiles at discharge. The daily injection frequency of injectable drugs at discharge were also compared between groups.

**Results:**

No specific trend of underlying diseases was observed between the non-Dula and Dula groups. The proportion of patients previously administered with GC pulse therapy was comparable between the two groups. No significant differences were observed between groups, in the starting maintenance GC dose, GC dose at pretreatment of Dula and discharge, and cumulative GC dose during the observation. Six-point blood glucose levels at pretreatment and discharge were comparable between the two groups. However, daily injection frequency of injectable drugs and insulin dose were significantly lower in the Dula group than that in the non-Dula group. No differences were observed in the number of hypoglycemic events, the elevation of serum pancreatic enzyme levels, or gastrointestinal adverse events.

**Conclusion:**

These findings suggest that Dula could provide glycemic control while reducing the insulin dose and injection frequency in inpatients with GC-induced hyperglycemia. The occurrence of adverse events such as gastrointestinal symptoms and hypoglycemia did not increase in the Dula-treated patients compared to those not treated, suggesting its safety.

## Key points

***Why carry out this study?***

*Multiple insulin injections is often necessary for glycemic control of glucocorticoid (GC)-induced hyperglycemia. A substitute insulin injection therapy is desired in patients with GC-induced hyperglycemia.*

***What was learned from the study?***

*A long-acting glucagon-like peptide 1 receptor agonist dulaglutide could provide glycemic control while reducing the insulin dose and injection frequency in the inpatient care for GC-induced hyperglycemia.*

## Background

Glucocorticoids (GCs) are one of the hormones produced in the adrenal cortex with an immunosuppressive action. They are widely used to treat autoimmune diseases, and nephrotic syndrome, and in organ transplantation, among other therapeutic applications. However, 2–30% of patients treated with GCs develop GC-induced diabetes mellitus [1, 2], and GC further promotes hyperglycemia in most patients with type 2 diabetes (T2DM). Risk factors for new-onset hyperglycemia during GC therapy are thought to be the same as those for other patients, including family history of DM, advanced age, obesity, and history of gestational DM [3].

GC-induced hyperglycemia is caused by the development of insulin resistance and beta-cell dysfunction [4–6]. Elevated postprandial blood glucose is a major characteristic of GC-induced hyperglycemia [7], and treatment with multiple insulin injections is often necessary for glycemic control [8]. This degrades the quality of life (QOL) in patients with DM [9, 10]. Thus, a substitute for insulin injection therapy is desirable in patients with GC-induced hyperglycemia.

Dulaglutide (Dula) is a long-acting glucagon-like peptide-1 receptor agonist (GLP-1RA) administered once weekly. A dose of 0.75 mg per week has been approved since 2015 for the treatment of T2DM in Japan. Treatment satisfaction is reportedly higher in patients injected with Dula than those injected daily with other GLP-1RA drugs according to the Diabetes Therapy-Related Quality of Life questionnaire in patients with T2DM [11].

It has been shown that GC promotes hyperglycemia in healthy subjects by impairing endogenous GLP-1 action [12]. Furthermore, a GLP-1RA, exenatide, has been reported to prevent GC-induced glucose intolerance by improving islet-cell dysfunction [13]. Therefore, Dula administration is suggested to effectively counteract these GC actions and GC-induced hyperglycemia, contributing to lower insulin injection frequency.

Therefore, this study aimed to retrospectively assess whether Dula could safely improve GC-induced hyperglycemia and lower insulin injection frequency and dose.

## Methods

### Patients

Investigators for this study collected the data of hospitalized patients diagnosed with GC-induced hyperglycemia (aged ≥20 years) as follows. First, all available hospitalized patients diagnosed with GC-induced hyperglycemia treated with 0.75 mg of Dula per week in the Third Department of Internal Medicine (Department of Endocrinology and Diabetes, Nephrology, and Collagen Diseases) at University of Yamanashi Hospital were reviewed and collected. As a result, 38 patients were included (Dula group). Second, data of control cases, which had most recent GC-induced hyperglycemia treated without Dula (non-Dula group) was collected until the same sample size (*n* = 38) as the Dula group. The need for informed consent was waived by the institutional review board of the University of Yamanashi (#1996), in view of the retrospective and observational nature of the study. An opt-out approach was used with the disclosure of website (https://www.med.yamanashi.ac.jp/rinri/ippan.html).

GC-induced hyperglycemia was defined as an elevation of blood glucose (fasting glucose level of ≥126 mg/dL or postprandial glucose level ≥ 200 mg/dL) during GC treatment including methylprednisolone pulse therapy. Patients with a history of T1DM, with concurrent malignancies, or who were being treated with insulin or a GLP-1 receptor agonist, were excluded. GC treatment, including methylprednisolone pulse therapy, had been newly initiated post-hospitalization in all patients.

Six-point blood glucose levels (before and 2 h after each meal) were obtained in all patients from the initiation of GC treatment including methylprednisolone pulse therapy. During pulse therapy, all 76 patients were treated with basal-bolus insulin, without Dula. Dula treatment was initiated from the beginning of GC treatment with the maintenance dose. All additional medications were allowed during the GC treatment based on the physicians’ discretion. Dipeptidyl peptidase-4 (DPP4) inhibitors were discontinued at the beginning of Dula treatment.

### Data collection

**Fig. 1:**
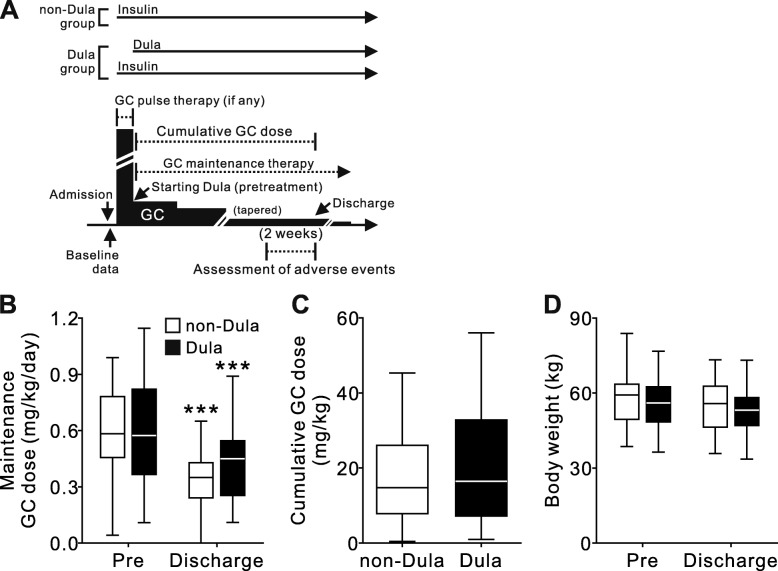
Changes in GC dose and body weight at pretreatment and discharge. **a** Timing of data collection, and treatment course of non-Dula and Dula groups in this study. **b** GC maintenance dose at pretreatment and discharge, and (**c**) cumulative GC dose during the observation. **d** Bodyweight changes at pretreatment and discharge. Data are expressed as Tukey’s box-and-whisker plots. Pre, pretreatment. GC, glucocorticoid. *** *p* < 0.001 vs. pretreatment of the same group.

The timings of data collection are summarized in Fig. 1a. Baseline characteristics and patient data were obtained at the beginning of GC treatment including GC pulse therapy. Six-point blood glucose profiles obtained using the OneTouch Verio IQ (LifeScan Japan, Inc., Japan) were assessed at the beginning of Dula treatment (defined as “pretreatment”), which is similar to the beginning of the maintenance dose of the GC therapy, and at discharge. The observation period was defined as from pretreatment to discharge, and the cumulative GC dose was calculated. The daily injection frequency of injectable drugs was assessed at maximal insulin dose and discharge. The injection frequency of Dula was calculated as 0.14 per day. The insulin dose was obtained at maximal insulin dose and discharge. Patients were also evaluated based on the dosage of oral GC converted to prednisolone (per kg of body weight).

### Outcome and adverse events

The primary outcome was glycemic control which was compared between Dula and non-Dula groups and assessed using six-point blood glucose profiles at discharge. The daily injection frequency of injectable drugs and timing of oral hypoglycemic agent administration were also assessed at pretreatment and dischage. Hypoglycemic and severe hypoglycemic episodes were defined as blood glucose levels of < 70 or < 54 mg/dl, respectively. Adverse events including gastrointestinal symptoms, hypoglycemic episodes, and elevated pancreatic enzymes were assessed during the 2 weeks before discharge.

### Statistical analysis

Continuous variables were reported as mean values ± one standard error or as median and interquartile range if not normally distributed, whereas categorical variables were reported as numbers and percentages. Differences between the Dula and non-Dula groups were tested using the Student’s t-test for unpaired data that demonstrated normality once (Kolmogorov–Smirnov test), otherwise, a nonparametric test (Mann–Whitney U-test) was used. Wilcoxon matched-pair signed-rank tests (two-tailed) were used to analyze each pairwise comparison within each group. Data are expressed as Tukey’s box-and-whisker plots. Categorical variables were analyzed using the χ2 test or Fisher’s exact test as necessary. Statistical analysis was performed using Microsoft Excel 2016 (Microsoft, Redmond, WA, USA) and Easy R (EZR; Saitama Medical Center, Jichi Medical University, Saitama, Japan). A *p*-value of < 0.05 was considered to indicate a statistically significant difference.

## Results

At baseline, the differences between the two groups in age, gender, weight, body mass index (BMI), history and duration of DM, blood pressure, fasting blood glucose, Hemoglobin A1-c (HbA1c), serum and urinary C-peptide, lipid profile, renal and liver function, and serum amylase and lipase levels, were not significant (Table 1).

**Table 1:** Patient characteristics

	Units	Non-Dula (*n* = 38)	Dula (*n* = 38)	*p*
Age	Year	68 ± 14	71 ± 9	0.21
Female	n (%)	18 (47)	17 (45)	0.82
Weight	kg	58 ± 10	56 ± 11	0.50
BMI	kg/m^2^	23.2 ± 3.7	21.9 ± 5.3	0.26
History of diabetes	n (%)	24 (63)	23 (61)	0.82
Duration of diabetes	Year	0 [0–14]	0 [0–11]	0.74
Observation period	Day	31 [19–49]	30 [16–48]	0.99
Underlying disease	n (%)			
Autoimmune disease		18 (47)	17 (45)	0.82
Kidney disease		8 (21)	6 (16)	0.55
Dermatosis		4 (11)	5 (13)	0.72
Pulmonary disease		3 (8)	4 (11)	0.69
Hematologic disease		2 (5)	1 (3)	0.56
Hepatopathy		2 (5)	2 (5)	1.00
Neurological disease		1 (3)	3 (8)	0.30
Diet therapy	kcal/day	1571 ± 221	1498 ± 179	0.14
SBP	mmHg	124 ± 16	122 ± 16	0.46
DBP	mmHg	72 ± 11	72 ± 10	0.99
HbA1c	%	7.6 ± 2.0	7.6 ± 1.9	0.99
FBG	mg/dL	156 [111–192]	177 [116–238]	0.27
Serum CPR	ng/mL	2.6 [1.5–4.1]	2.6 [1.5–4.0]	0.98
Urinary CPR	μg/day	46 [13–95]	33 [11–49]	0.31
TG	mg/dL	121 [91–167]	107 [86–156]	0.43
LDL-C	mg/dL	116 [101–151]	97 [79–145]	0.10
HDL-C	mg/dL	48 [39–48]	46 [38–59]	0.83
Serum creatinine	mg/dL	0.93 [0.65–2.24]	0.79 [0.57–1.28]	0.21
eGFR	mL/min /1.73 m^2^	49 [23–74]	60 [45–85]	0.14
ALT	U/L	24 [14–39]	16 [11–25]	0.11
AST	U/L	23 [16–30]	19 [14–27]	0.27
Amylase	U/L	71 [43–102]	69 [57–104]	0.50
Lipase	U/L	32 [18–60]	39 [27–48]	0.72

*BMI* Body mass index, *SBP* Systolic blood pressure, *DBP* Diastolic blood pressure, *FBG* Fasting blood glucose, *CPR* C-peptide, *TG* Triglyceride, *LDL-C* Low-density lipoprotein cholesterol, *HDL-C* High-density lipoprotein cholesterol, *eGFR* Estimated glomerular filtration rate, *ALT* Alanine transaminase, *AST* Aspartate aminotransferase

Differences in the observation period and the frequency of underlying diseases indicated for GC treatment, were also not significant. The starting maintenance GC dose, dose of GC at discharge, and cumulative GC dose during the observation period were comparable between the two groups (Fig. 1b and c). The number of patients without history of diabetes was 14 and 15 in non-Dula and Dula groups, respectively (Table 1). Among them, 5 patients in non-Dula group and 6 patients in Dula group, whose proportion was not statistically significant different between two groups, did not meet the criteria for diabetes based on their baseline glucose profiles (data not shown). Seven patients in the non-Dula group and six in the Dula group had received pulse therapy after admission followed by GC treatment with maintenance dose, and no significant differences were observed in their proportions (data not shown). Body weights at pretreatment and discharge were comparable between the two groups (Fig. 1c). No significant weight changes were observed in both groups.

A total of 20 patients in the non-Dula group and 23 in the Dula group were treated with oral hypoglycemic agents at pretreatment, and no significant differences were observed (Table 2). At pretretment, oral α-glucosidase inhibitors (α-GI) were more frequently administered to patients in the Dula group, however, the difference did not reach statistical significance.

**Table 2:** Use of oral antihyperglycemic drugs at pretreatment and discharge

	non-Dula (*n* = 38)		Dula (*n* = 38)	
	Pre	Discharge	Pre	Discharge
Type of OADs, n (%)	20 (53)	25 (66)	23 (61)	23 (61)
DPP-4i, n (%)	18 (47)	22 (58)	20 (53)	0 (0)***^, ###^
BG, n (%)	4 (11)	6 (16)	8 (21)	9 (24)
α-GI, n (%)	2 (5)	5 (13)	7 (18)	11 (29)
Glinide, n (%)	0 (0)	8 (21)**	2 (5)	11 (29)**
SU, n (%)	2 (5)	0 (0)	3 (8)	1 (3)
SGLT-2i, n (%)	0 (0)	0 (0)	3 (8)	2 (5)
TZD, n (%)	0 (0)	0 (0)	0 (0)	0 (0)

*OADs* Oral antihyperglycemic drugs, *DPP-4i* Dipeptidyl peptidase-4 inhibitors, *BG* Biguanides, *α-GI* α-glucosidase inhibitors, *SU* Sulfonylurea, *SGLT-2i* Sodium-glucose cotransporter-2 inhibitors, *TZD* Thiazolidinediones. *Pre* Pretreatment. ** *p* < 0.01, *** *p* < 0.001 vs. pretreatment. ^###^*p* < 0.001 vs. non-Dula group

**Fig. 2:**
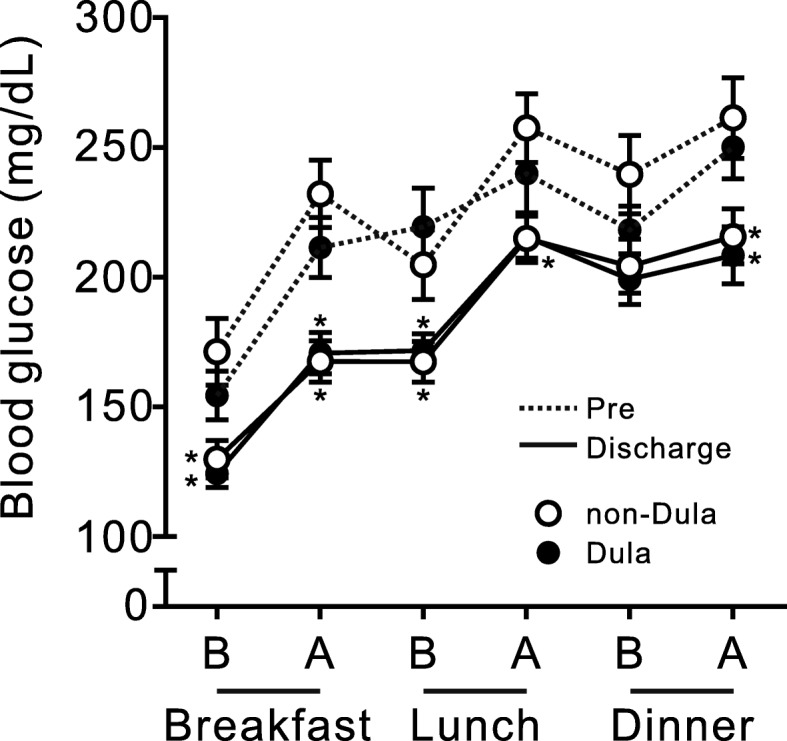
Six-point blood glucose levels at pretreatment and discharge. Six-point blood glucose levels at pretreatment and discharge. B, before meal; A, after meal. Pre, pretreatment. * *p* < 0.05 vs. pretreatment of the same group.

**Fig. 3:**
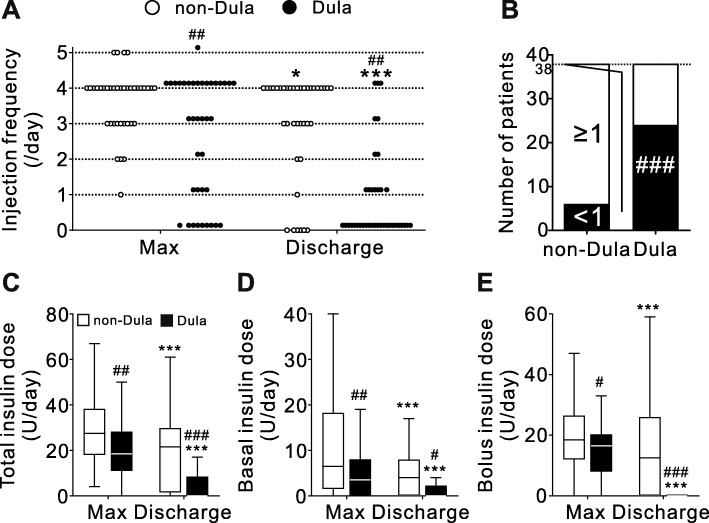
Daily injection frequency of injectable drugs and insulin dose at pretreatment and discharge. **a** Daily injection frequency of injectable drugs at maximal insulin dose (Max) and discharge. Dotted lines indicate integral numbers. **b** The number of patients whose daily injection frequency of injectable drugs was less than once per day (<1, black bar) and once per day or more (≥1, white bar). **c** Total insulin dose at Max and discharge, **d** basal insulin dose at Max and discharge, and (**e**) bolus insulin dose at Max and discharge. Daily injection frequency of Dula was calculated as 0.14 per day. Data are expressed as Tukey’s box-and-whisker plots. Pre, pretreatment. Max, maximal insulin dose. * *p* < 0.05 and *** *p* < 0.001 vs. Max of the same group. # *p* < 0.05, ## *p* < 0.01, and ### *p* < 0.001 vs. non-Dula group.

Six-point blood glucose levels at pretreatment and discharge were comparable between the two groups (Fig. 2). In both groups, the daily frequency of injectable drug administration decreased from when the insulin dose was maximal, to discharge (Fig. 3a). However, in the Dula group, the frequency was significantly lower when the insulin dose was maximal and at discharge than in the non-Dula group (Fig. 3a). At discharge, the proportion of patients with an injection frequency less than once per day was significantly higher in the Dula group than in the non-Dula group (Fig. 3b). Whereas the total, basal, and bolus insulin dose at discharge were significantly reduced in both groups compared to when the insulin dose was maximal, the dose was significantly lower in the Dula group than the non-Dula group (Fig. 3c–e). The proportion of patients with bolus insulin injection at breakfast, lunch, and dinner at discharge was significantly lower in Dula group than that of non-Dula group (Supplementary Figure 1).

No differences between the two groups were found in the number of patients with blood glucose levels of < 70 mg/dL during the 2 weeks before discharge, however, one patient in the Dula group experienced severe hypoglycemia (Table 3). In all patients with blood glucose levels of < 70 mg/dL, hypoglycemia was treated with oral glucose therapy and was not associated with other adverse events. In both groups, no elevation in amylase and lipase levels at discharge were observed, and no significant differences in frequency of constipation, nausea, vomiting, anorexia, diarrhea, satiety, and rash were observed between the two groups.

**Table 3:** Adverse events during the 2 weeks before discharge

	non-Dula (n = 38)	Dula (n = 38)	*p*
Hypoglycemic events, n (%)			
Severe Hypoglycemia	0 (0)	1 (3)	0.53
hypoglycemia	7 (18)	5 (13)	0.31
Elevation of amylase, n (%)	2 (5)	2 (5)	1.00
Elevation of lipase, n (%)	3 (8)	1 (3)	0.30
Constipation, n (%)	12 (32)	14 (37)	0.20
Nausea, n (%)	1 (3)	3 (8)	0.23
Vomiting, n (%)	0 (0)	1 (3)	0.31
Anorexia, n (%)	3 (8)	4 (11)	0.60
Diarrhea, n (%)	2 (5)	0 (0)	0.15

## Discussion

The principal finding of this study is that Dula improved GC-induced hyperglycemia during inpatient care and was associated with a lower injection frequency and insulin dose than observed in patients not treated with Dula. Although a case report has been published showing that Dula improves GC-induced diabetes mellitus [14], this study has shown the drug’s effectiveness in the treatment of GC-induced hyperglycemia in a larger sample.

Current management strategies provide insufficient guidance for glycemic control in individuals treated with GCs and GC-induced hyperglycemia is usually managed with insulin. It has been reported that insulin injection therapy was required in up to 50% of renal transplant recipients treated with high-dose GC [15]. In patients initially treated with a medium-high dose of GC in the morning, as were the majority of patients in this study, the increased blood glucose level is detected mainly in the postprandial state and afternoon [16]. Consequently, an increased frequency of injections due to bolus and basal-bolus insulin treatment is often inevitable [17]. However, increased insulin injection frequency is reportedly associated with decreased patient QOL [9, 10]. A previous report using the Diabetic Treatment Burden Questionnaire (DTBQ), a patient-administered questionnaire for measuring the burden of pharmacotherapy of T2DM, showed that one injection per week is associated with a lower injection burden than one or more injections per day [18]. In the present study, the proportion of patients with an injection frequency less than once per day was significantly higher in the Dula group than in the non-Dula group. The result suggests that, in GC-induced hyperglycemia, Dula may contribute to increase patients’ QOL by reducing injection frequency.

Our observation suggests that Dula can be a promising option for improving patient QOL, for the treatment of GC-induced hyperglycemia in inpatient care.

The mechanisms that cause GC-related hyperglycemia are multifactorial and include stimulation of hepatic gluconeogenesis, inhibition of glucose uptake by adipose tissue, and alteration of receptor and post-receptor functions [4, 19, 20]. Moreover, GCs are also reported to impair endogenous incretin actions [12, 13]. Since the modes of action of insulin and GLP-1 can antagonize these GC-induced changes in glucose metabolism [21], it is conceivable that Dula can partly substitute insulin’s glucose-lowering effect, reducing the injection frequency.

This study showed that the prevalence of adverse effects was comparable between the Dula and non-Dula groups. Dula (0.75 mg per week, the only approved dose in Japan) has been reported to increase gastrointestinal adverse events including nausea, constipation, and diarrhea compared to insulin glargine in Japanese patients in a phase 3 study [22, 23]. It is conceivable that the gastrointestinal adverse effects of Dula may be partly compensated for by a GC-induced increase in appetite. In contrast to our observation that the incidence of hypoglycemia in the Dula group was not lower than that in the non-Dula group, the phase 3 study showed that the incidence of hypoglycemia was significantly lower in the Dula group compared with glargine [22]. It may be because tight glycemic control could not be achieved in this study. The long-term safety of Dula in GC-induced hyperglycemia should be evaluated further.

The majority of patients in the Dula group were also treated with insulin, suggesting that Dula cannot suppress GC-induced hyperglycemia without insulin. Both at maximal dose of insulin and discharge, the dose and injection frequency were more markedly reduced in the Dula group than in the non-Dula group. In addition, the frequency of administration of oral antidiabetic drugs for postprandial hyperglycemia, such as α-GI and glinide, was comparable between the two groups at discharge. This suggests that patients on Dula treatment needed less additional bolus insulin to control the postprandial glucose increase caused by the GCs than those not administered Dula. Dula acts by stimulating insulin secretion and reducing glucagon levels in a glucose-dependent manner in both fasting and postprandial states, resulting in reductions of fasting and postprandial glucose levels at 0.75 mg per week [22]. However, because GC-induced hyperglycemia is detected mainly in the postprandial state, it was possible that the effect of Dula on decreasing fasting glucose levels did not fully develop. Consequently, the dose and injection frequency of basal insulin were less affected by Dula than those of bolus insulin. In addition, when the GC dose is being tapered off after chronic treatment, concomitant use of Dula from the initiation of GC therapy may allow further reduction, and possibly, withdrawal of the bolus insulin injection. The long-term treatment sustainability of Dula and its effect on insulin injection frequency in this phase of chronic GC treatment require further research.

Our study has limitations. First, the selection criteria for Dula treatment was not standardized. Therefore the selection might be subject to the physicians’ unmeasurable biases. For example, the physicians might select Dula for the patients who have poor treatment adherence, or for social reasons. Although no significant differences in patient characteristics were observed, these biases could occur as a result of retrospective analysis based on clinical practice. Second, the treatment protocol of GC-induced hyperglycemia was not standardized in the Dula and non-Dula groups. Third, the long-term efficacy and safety were not assessed in this study. Longer and more controlled prospective comparisons will be needed to provide evidence to support the effectiveness of Dula for the treatment of GC-induced hyperglycemia. Fourth, the use of Dula was currently off-label for the GC-induced hyperglycemia in Japan.

In this study, approximately 40% of all subjects did not have a history of DM. A report has shown that new-onset hyperglycemia occurs in 32.3% of non-diabetic patients treated with GC and that the risk of developing DM reportedly increases 10.3-fold with GC administration at a prednisolone equivalent dose of ≥30 mg/day in non-diabetic patients [2]. Risk factors for new-onset hyperglycemia during GC therapy are thought to be the same as those for other patients, including a family history of DM, increased age, obesity, and history of gestational DM [3]. Then, screening should be performed even in those taking corticosteroids at low doses. Measurement of lunchtime postprandial glycemia offers the greatest diagnostic sensitivity, especially when intermediate-acting GCs are administered in a single morning dose.

## Conclusion

This study suggests that Dula could provide glycemic control while reducing the insulin dose and injection frequency required during the inpatient care of patients with GC-induced hyperglycemia. Adverse events such as gastrointestinal symptoms and hypoglycemia were not increased in Dula-treated patients, suggesting that it may be safely used.

## Supplementary information

**Additional file 1: Figure S1.** Injection frequency of bolus insulin at each meal. The number of patients with (black bar) or without (white bar) bolus insulin injection at each meal at maximal insulin dose (Max) and discharge. * *p* < 0.05 and ** *p* < 0.01 vs. Max of the same group. # p < 0.05, ## p < 0.01, and ### *p* < 0.001 vs. non-Dula group.

## Data Availability

Datasets obtained and/or analyzed in this study are available from the corresponding author on reasonable request.

## Abbreviations

GC: Glucocorticoid
Dula: Dulaglutide
T2DM: Type 2 diabetes
QOL: Quality of life
GLP-1RA: Glucagon-like peptide-1 receptor agonist
DPP-4i: Dipeptidyl peptidase-4 inhibitors
BMI: Body mass index
HbA1c: Hemoglobin A1-c
α-GI: α-glucosidase inhibitors
DTBQ: The Diabetic Treatment Burden Questionnaire
ICMJE: The International Committee of Medical Journal Editors
