# Supplementary material for: Dulaglutide improves glucocorticoid-induced hyperglycemia in inpatient care and reduces dose and injection frequency of insulin
Source: BMC Endocr Disord. 2020 May 7;20:58. doi: 10.1186/s12902-020-0542-5 (PMC7203793; doi:10.1186/s12902-020-0542-5)

Supplementary Information

Dulaglutide Improves Glucocorticoid-induced Hyperglycemia in Inpatient Care and Reduces Dose and Injection Frequency of Insulin

Hiroyuki Uchinuma, Masashi Ichijo, Noriyuki Harima and Kyoichiro Tsuchiya

Department of Diabetes and Endocrinology, University of Yamanashi Hospital, 1110 Shimokato, Chuo, Yamanashi, 4093898, Japan.

SUPPLEMENTARY FIGURE LEGEND

Supplementary Figure 1. Injection frequency of bolus insulin at each meal. The number of patients with (black bar) or without (white bar) bolus insulin injection at each meal at maximal insulin dose (Max) and discharge. * $p < 0.05$ and ** $p < 0.01$ vs. Max of the same group. # $p < 0.05$, ## $p < 0.01$, and ### $p < 0.001$ vs. non-Dula group.

Supplementary Figure 1

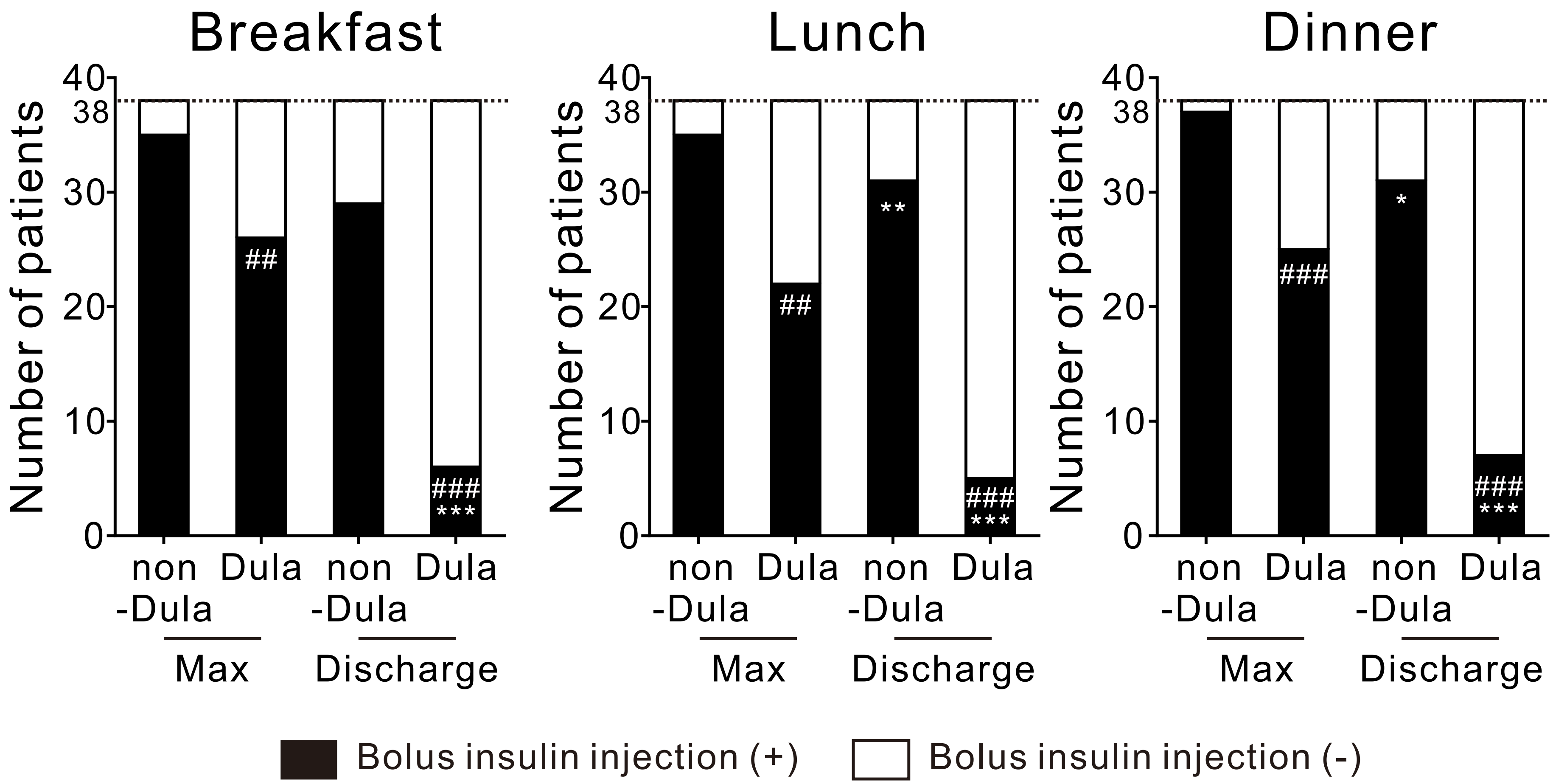

Supplement: Supplementary file 1 — Additional file 1: Figure S1. Injection frequency of bolus insulin at each meal. The number of patients with (black bar) or without (white bar) bolus insulin injection at each meal at maximal insulin dose (Max) and discharge. * p < 0.05 and ** p < 0.01 vs. Max of the same group. # p < 0.05, ## p < 0.01, and ### p < 0.001 vs. non-Dula group. [file 12902_2020_542_MOESM1_ESM.pdf]
